# The CRISPR-Cas System Is Involved in OmpR Genetic Regulation for Outer Membrane Protein Synthesis in *Salmonella* Typhi

**DOI:** 10.3389/fmicb.2021.657404

**Published:** 2021-03-29

**Authors:** Liliana Medina-Aparicio, Sarahí Rodriguez-Gutierrez, Javier E. Rebollar-Flores, Ángel G. Martínez-Batallar, Blanca D. Mendoza-Mejía, Eira D. Aguirre-Partida, Alejandra Vázquez, Sergio Encarnación, Edmundo Calva, Ismael Hernández-Lucas

**Affiliations:** ^1^Departamento de Microbiología Molecular, Instituto de Biotecnología, Universidad Nacional Autónoma de México, Cuernavaca, Mexico; ^2^Centro de Ciencias Genómicas, Universidad Nacional Autónoma de México, Cuernavaca, Mexico

**Keywords:** CRISPR-Cas, porin regulation, *Salmonella* Typhi, OmpR, outer membrane proteins

## Abstract

The CRISPR-Cas cluster is found in many prokaryotic genomes including those of the Enterobacteriaceae family. *Salmonella enterica* serovar Typhi (*S*. Typhi) harbors a Type I-E CRISPR-Cas locus composed of *cas3*, *cse1*, *cse2*, *cas7*, *cas5*, *cas6e*, *cas1*, *cas2*, and a CRISPR1 array. In this work, it was determined that, in the absence of *cas5* or *cas2*, the amount of the OmpC porin decreased substantially, whereas in individual *cse2*, *cas6e*, *cas1*, or *cas3* null mutants, the OmpF porin was not observed in an electrophoretic profile of outer membrane proteins. Furthermore, the LysR-type transcriptional regulator LeuO was unable to positively regulate the expression of the quiescent OmpS2 porin, in individual *S*. Typhi *cse2*, *cas5*, *cas6e*, *cas1*, *cas2*, and *cas3* mutants. Remarkably, the expression of the master porin regulator OmpR was dependent on the Cse2, Cas5, Cas6e, Cas1, Cas2, and Cas3 proteins. Therefore, the data suggest that the CRISPR-Cas system acts hierarchically on OmpR to control the synthesis of outer membrane proteins in *S*. Typhi.

## Introduction

Microorganisms are constantly exposed to multiple viral infections and have developed many strategies to survive phage attack and invasion by foreign DNA. One such strategy is the CRISPR-Cas bacterial immunological system (Barrangou et al., 2007). This system is classified according to the presence of signature Cas proteins (Makarova et al., 2011, 2015). The hallmark of the CRISPR-Cas Type I system is the presence of the endonuclease Cas3. This protein is involved in cleavage of exogenous target nucleic acids (Sinkunas et al., 2011; Westra et al., 2012). The Type II system requires Cas9 and a trans-activating CRISPR RNA (tracrRNA) for DNA recognition and degradation (Deltcheva et al., 2011). The Type III system uses the RAMP proteins and Cas10 nuclease to silence the invader (Samai et al., 2015; Elmore et al., 2016).

In the Enterobacteriaceae family, the Type I CRISPR-Cas is the predominant system. The analysis of 228 enterobacterial genomes, corresponding to 38 genera, showed that 55% present, at least, one Type I CRISPR-Cas system (Medina-Aparicio et al., 2018). In the *Salmonella* genus, two CRISPR arrays (CRISPR1 and CRISPR2) have been identified, and only CRISPR1 is associated with a Type I-E set of *cas* genes (Touchon and Rocha, 2010). In 35 of 38 *Salmonella* genomes analyzed so far, the Type I-E CRISPR-Cas system was present, whereas *S. enterica* serovars Pullorum S06004, Javiana and Paratyphi B did not have any *cas* genes (Medina-Aparicio et al., 2018).

**Figure 1:**
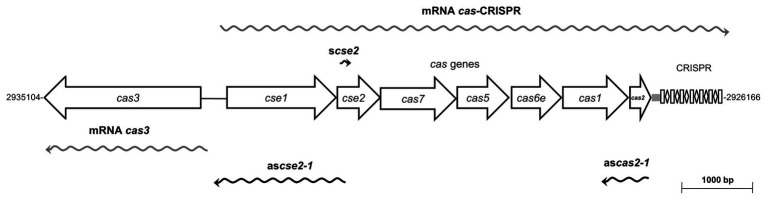
Transcriptional organization of the Type I-E CRISPR-Cas system of *Salmonella enterica* serovar Typhi IMSS-1. The CRISPR-Cas system is composed of eight *cas* genes (*cas3* and *cse1-cse2-cas7-cas5-cas6e-cas1-cas2*), a leader of 84 bp and the CRISPR1 array, containing seven 29-bp repeats and six 32-bp spacers. Five transcriptional units are transcribed from this locus, wavy gray arrows represent mRNAs of *cas*-CRISPR operon and *cas3*, whereas the RNA s*cse2*, the antisense as*cse2-1* and as*cas2-1* are shown as wavy black arrows. The *Salmonella* Typhi ΔCRISPR-*cas* strain is devoided of the entire Type I-E CRISPR-Cas system (from *cas3* to CRISPR locus).

*Salmonella* Typhi IMSS-1 harbors a Type I-E CRISPR-Cas cluster composed of *cas3*, *cse1-cse2-cas7-cas5-cas6e-cas1-cas2*, an 84-bp leader sequence, seven 29-bp repeats and six 32-bp spacers with no homologous sequences reported in the DDBJ data bank (Medina-Aparicio et al., 2011). This locus contains five transcriptional units, two of them are the *cse1-cse2-cas7-cas5-cas6e-cas1-cas2-*CRISPR (*cas*-CRISPR operon) and s*cse2* (sense *cse2* RNA), are transcribed from the sense strand, whereas as*cse2-1* (antisense RNA of *cse2* to *cse1*) and as*cas2-1* (antisense RNA of *cas2* to *cas1*) are present on the antisense strand (Medina-Aparicio et al., 2017). Additionally, the *S*. Typhi *cas3* gene is transcribed as an independent unit divergent from the *cas*-CRISPR operon (Figure 1). The transcription of the *cse1-cse2-cas7-cas5-cas6e-cas1-cas2*-CRISPR polycistronic mRNA is induced by LeuO and negatively regulated by H-NS and Lrp (Hernández-Lucas et al., 2008; Medina-Aparicio et al., 2011). The role of H-NS in silencing the expression of the *cas3* and as*cse2-1* transcriptional units has also been demonstrated. The transcriptional activities of the five transcriptional units present in the *S.* Typhi CRISPR-Cas locus are induced by basic pH (Medina-Aparicio et al., 2017).

Relevant work on the regulation and the signals that induce the *S.* Typhi CRISPR-Cas system has been reported by our group (Hernández-Lucas et al., 2008; Medina-Aparicio et al., 2011, 2017). However, its biological function remains to be determined. In this regard, the low number of spacers in the CRISPR sequences, as well as their non-homology with bacteriophage and plasmid sequences, suggest that the CRISPR-Cas system does not provide an immune function in *Salmonella*. However, the conserved genetic organization of the *cas* genes in different *Salmonella* serovars is consistent with the system having a biological function in these bacteria (Touchon and Rocha, 2010; Shariat et al., 2015). In this work, it is reported that, in *S.* Typhi, CRISPR-Cas positively regulates OmpR, a two-component system regulator that induces the synthesis of the OmpC, OmpF, and OmpS2 porins. Additionally, it is demonstrated that the CRISPR-Cas system is involved in the resistance to bile salts and biofilm formation in *S.* Typhi.

## Materials and Methods

### Bacterial Strains, Plasmids, and Culture Conditions

The bacterial strains and plasmids used in this work are listed in Supplementary Table S1. *Salmonella* Typhi IMSS-1 (Puente et al., 1987) and *Escherichia coli* strains were grown aerobically at 37°C in LB (10 g tryptone, 5 g yeast extract, and 10 g NaCl per liter), MA (7 g nutrient broth, 1 g yeast extract, 2 ml glycerol, 3.75 g K_2_HPO_4_, and 1.3 g KH_2_PO_4_ per liter; Kawaji et al., 1979) or N-MM media [0.37 g KCl, 0.99 g (NH_4_)_2_SO_4_, 0.087 g K_2_SO_4_, 0.14 g KH_2_PO_4_, 0.019 g MgCl_2_, 1 g casamino acids, 5 ml glycerol, and 100 mM of Tris-HCl (pH 7.5) per liter] (Deiwick et al., 1999). When required, the following antibiotics were added: kanamycin (Km), 30 μg ml^−1^; tetracycline (Tc), 12 μg ml^−1^, and ampicillin (Ap), 200 μg ml^−1^.

### DNA Manipulations

Plasmid and genomic DNA isolations were carried out according to published protocols (Sambrook et al., 1989). Primers for PCR amplifications were provided by the Oligonucleotide Synthesis Facility at our Institute (Supplementary Table S2). Restriction enzymes, ligase, nucleotides, and polymerases were acquired from New England Biolabs, Invitrogen, or Thermo Scientific. For sequencing, double-stranded DNA was purified with the High Pure Plasmid Isolation Kit (Roche) and sequenced with an automatic Perkin Elmer/Applied Biosystems 377-18 system.

### Site-Directed Mutagenesis

The *Salmonella* mutants were obtained by the one-step non-polar mutagenesis procedure (Datsenko and Wanner, 2000). The target gene was replaced with selectable antibiotic resistance gene markers. The resistance cassette was removed using the pCP20 plasmid. Each mutation was further characterized by sequencing to verify the authenticity of the deletion.

### Construction of Transcriptional Reporter Fusions

For transcriptional *cat* constructs, oligonucleotides (see Supplementary Table S2) were designed to amplify DNA fragments of different lengths from the *ompC*, *ompF*, *ompS2*, and *ompR* regulatory regions. PCR products were double-digested with *Bam*HI-*Kpn*I and ligated into pKK232-8 or pKK232-9 (Supplementary Table S1), which contain the promoterless *cat* gene. All constructs were sequenced to verify the correct DNA sequence of the PCR fragments.

### CAT Assays

To determine the expression of the *cat* reporter gene mediated by the *S.* Typhi promoters, chloramphenicol acetyltransferase (CAT) assays were performed according to a previously published protocol (Martínez-Laguna et al., 1999). Briefly, *S.* Typhi strains harboring the reporters were grown in N-MM or MA to different optical densities (OD), and the latter medium was supplemented when required with Ap and Km, with or without IPTG (isopropyl-β-d-thiogalactopyranoside; 50 μM). Cells were harvested, centrifuged, washed with 0.8 ml of TDTT buffer (50 mM Tris-HCl, 30 μM DL-dithiothreitol, and pH 7.8), resuspended in 0.5 ml of TDTT, and sonicated on ice for 10-s intervals with 10-s rest periods until the extract was clear. The homogenate was centrifuged at 12,000 *g*/15 min, and the supernatant used for activity measurement. For CAT assays, 5 μl of each extract were added in duplicate to a 96-well enzyme-linked immunosorbent assay (ELISA) plate, followed by the addition of 0.2 ml of a reaction mixture containing 1 mM DTNB [5,5'-dithiobis (2-nitrobenzoic acid)], 0.1 mM acetyl-coenzyme A (acetyl-CoA), and 0.1 mM chloramphenicol in 0.1 M Tris-HCl, pH 7.8. The absorbance at 412 nm was measured every 5 s for 5 min using a Ceres 900 scanning auto reader and microplate workstation. The protein concentration of the cell extracts was obtained using the bicinchoninic acid (BCA) protein assay reagent (Pierce). Protein values and the mean rate of product formation by CAT were used to determine CAT-specific activity as micromoles per minute per milligram of protein.

### Preparation of Crude Cell Extracts for Two-Dimensional Gel Electrophoresis

*Salmonella* Typhi IMSS-1 and *S.* Typhi Δ*cas*-CRISPR harboring plasmid pFM*TrcleuO*-50 were grown in MA medium supplemented with Ap and IPTG (50 μM) to an optical density of 0.6 at 595 nm (OD_595_). *Salmonella* cultures (100 ml) were pelleted and washed with 1X phosphate-buffered saline (PBS). Cellular proteins were obtained by sonication at 24 kHz for 1 min in the on position and 1 min in the off position, for five cycles at 4°C using a Vibra Cell (Sonics, United States), in the presence of a protease inhibitor (Complete tablets; Roche Diagnostics GmbH, Mannheim, Germany). To further limit proteolysis, protein isolation was performed using phenol extraction (Hurkman and Tanaka, 1986). To solubilize proteins and to obtain completely denatured and reduced proteins, pellets were dried and resuspended as previously reported (Encarnación et al., 2005). Prior to electrophoresis, samples were mixed with 7 M urea, 2 M thiourea, 4% 3-[(3-choloamidopropyl)-dimethylammonio]-1-propanesulfonate (CHAPS; Roche Diagnostics GmbH, Germany), 2 mM tributylphosphine, 2% ampholytes, and 60 mM dithiothreitol.

### Two-Dimensional Gel Electrophoresis

Methods used for sample preparation, analytical two-dimensional gel electrophoresis (2-DGE), image analysis, and preparative 2-DGE were described previously (Encarnación et al., 2003). pH gradients were determined using a two-dimensional sodium dodecyl sulfate-polyacrylamide gel electrophoresis standard (Sigma, United States). For isoelectric focusing, 500 μg of total proteins were loaded. All gel experiments were repeated at least two times.

### In-Gel Digestion and Mass Spectrometry-Based Identification of Proteins

Selected spots from Coomassie blue-stained preparative one- or two-dimensional gels were excised manually and frozen at −70°C until use. Samples were prepared for mass spectrum analysis using a slight modification of a previously described procedure (Encarnación et al., 2005). Protein spots were destained, reduced, alkylated, and digested with trypsin (Promega, Madison, WI). Before the mass spectra of the peptide mixtures were obtained, the mixtures were desalted using a C_18_ Zip Tip (Millipore, Bedford, MA) according to the manufacturer’s recommendations. Mass spectra were determined using a Bruker Daltonics Autoflex (Bruker Daltonics, Billerica, MA) operated in the delayed extraction and reflectron mode. Spectra were externally calibrated using a peptide calibration standard (Bruker Daltonics 206095). Peptide mixtures were analyzed using a saturated solution of alpha-cyano-4-hydroxycinnamic acid in 50% acetonitrile-0.1% trifluoroacetic acid. Peak lists of the tryptic peptide masses were generated and searched against the NCBInr databases using the Mascot search program (Matrix Science, London, United Kingdom).^1^

### Preparation of Outer Membrane Proteins

Outer Membrane Proteins (OMPs) were isolated from *S.* Typhi IMSS-1 strains grown in N-MM to an OD_595_ of 0.6 and 1.3 according to previous protocols (Puente et al., 1995). Fifteen milliliter of each culture was harvested and centrifuged at 5,000 *g* for 10 min at 4°C. Cells were resuspended in 500 μl of 10 mM Na_2_HPO_4_ buffer (pH 7.2) and sonicated on ice until the suspensions were clear. Intact cells and debris were eliminated by centrifugation (15,000 *g*) for 2 min, and the supernatants were transferred to clean microcentrifuge tubes and membrane fractions were pelleted by centrifugation at 12,000 *g* for 1 h at 4°C. Inner membrane proteins were solubilized by resuspension in 500 μl of 10 mM Na_2_HPO_4_ buffer, pH 7.2, containing 2% Triton X-100 for 30 min at 37°C. After incubation, the samples were centrifuged at 12,000 *g* for 1 h at 4°C. The remaining outer membrane insoluble fraction was washed with 500 μl of 10 mM Na_2_HPO_4_, pH 7.2, centrifuged at 12,000 *g* for 1 h at 4°C, and finally resuspended in 50 μl 1X PBS, pH 7.4. OMP concentrations were determined by BCA assay (Thermo), and 15 μg of each sample was analyzed by SDS-12% polyacrylamide gel electrophoresis. One-dimensional OMP gels were visualized by staining with Coomassie brilliant blue.

### Western Blotting

For western blot experiments, *S.* Typhi wild-type strain and its derivatives were grown in N-MM to OD_595_ of 1.0 or MA medium to an OD_595_ of 0.6. The cultures were supplemented, when required, with Ap and IPTG (50 μM). Fifteen milliliter of each culture was harvested and centrifuged at 5,000 *g* for 8 min. The pellets were resuspended in 600 μl of 1X PBS and sonicated on ice for 12 min at intervals of 10-s with 5-s rest. Total protein concentration was determined by BCA assay (Thermo), and 80 μg of each sample was loaded on a 10% SDS polyacrylamide gel. Following electrophoresis, proteins were transferred to 0.45-μm-pore-size polyvinylidene difluoride membranes (Immobilon; Millipore) using the Trans-Blot SD system (Bio-Rad) according to a previously described procedure (Guadarrama et al., 2014). Membranes were blocked with 10% non-fat milk and incubated with anti-OmpR or anti-GroEL (StressGen) polyclonal antibodies. Then, they were washed with 1X PBS, 0.1% Tween 20. Immunodetection was performed with a 1:10,000 dilution of horseradish peroxidase-conjugated Anti-Rabbit antibody (Pierce) for polyclonal antibodies, and the Western Lightning Plus-ECL Chemiluminescence Reagent Kit (PerkinElmer). The membranes containing the proteins were exposed to Carestream X-OMAT LS films.

### Growth Evaluation in 5% Sodium Deoxycholate

*Salmonella* Typhi wild-type and the different mutant strains were grown 24 h in LB plates at 37°C. A bacterial colony was inoculated in liquid LB broth (5 ml) and grown for 16 h at 37°C/200 rpm. Then, 50 ml of LB broth supplemented with 5% sodium deoxycholate (Sigma Chemical, St. Louis, MO) were inoculated with the pre-inoculum to give an initial OD at 595 nm of 0.02. The cultures were incubated at 37°C/200 rpm during 15 h with OD_595_ measurements being done every 2 h.

### Microtiter Dish Biofilm Formation Assay

The quantification of biofilm formation was performed following a previous established protocol (O’Toole, 2011). Briefly, bacterial cells were grown overnight in LB broth (5 ml) at 37°C/200 rpm. Cells were diluted 1:100 in fresh LB without NaCl for stimulates biofilm production. One hundred microliter of this dilution was added per well in a 96-well polystyrene microtitre plate (Costar Cat. No. 3599, flat bottom with lid). Six replicate wells were prepared for each strain. Microtitre plates were incubated at 30°C for 24 h. Total bacterial growth was measured at OD_600_, using a GloMax®-Muti Detection System (Promega). The planktonic cells were then discarded, and the plate was washed three times with water. The remaining biofilm was fixed with 200 μl per well of methanol (100%) and stained with a 0.2% solution of crystal violet in water. After incubation at room temperature for 10 min, the plates were rinsed three times with water. The dye was solubilized by adding 125 μl of 33% acetic acid to each well and incubated the microtiter plate at room temperature for 15 min. Finally, the OD_560_ was determined with the microplate reader. The amount of formed biofilm is reported as the ratio of the OD_560_/OD_600_ values (Oropeza et al., 2015).

## Results

### CRISPR-Cas Is Fundamental for the Synthesis of Major and Quiescent Outer Membrane Proteins in *Salmonella* Typhi

**Figure 2:**
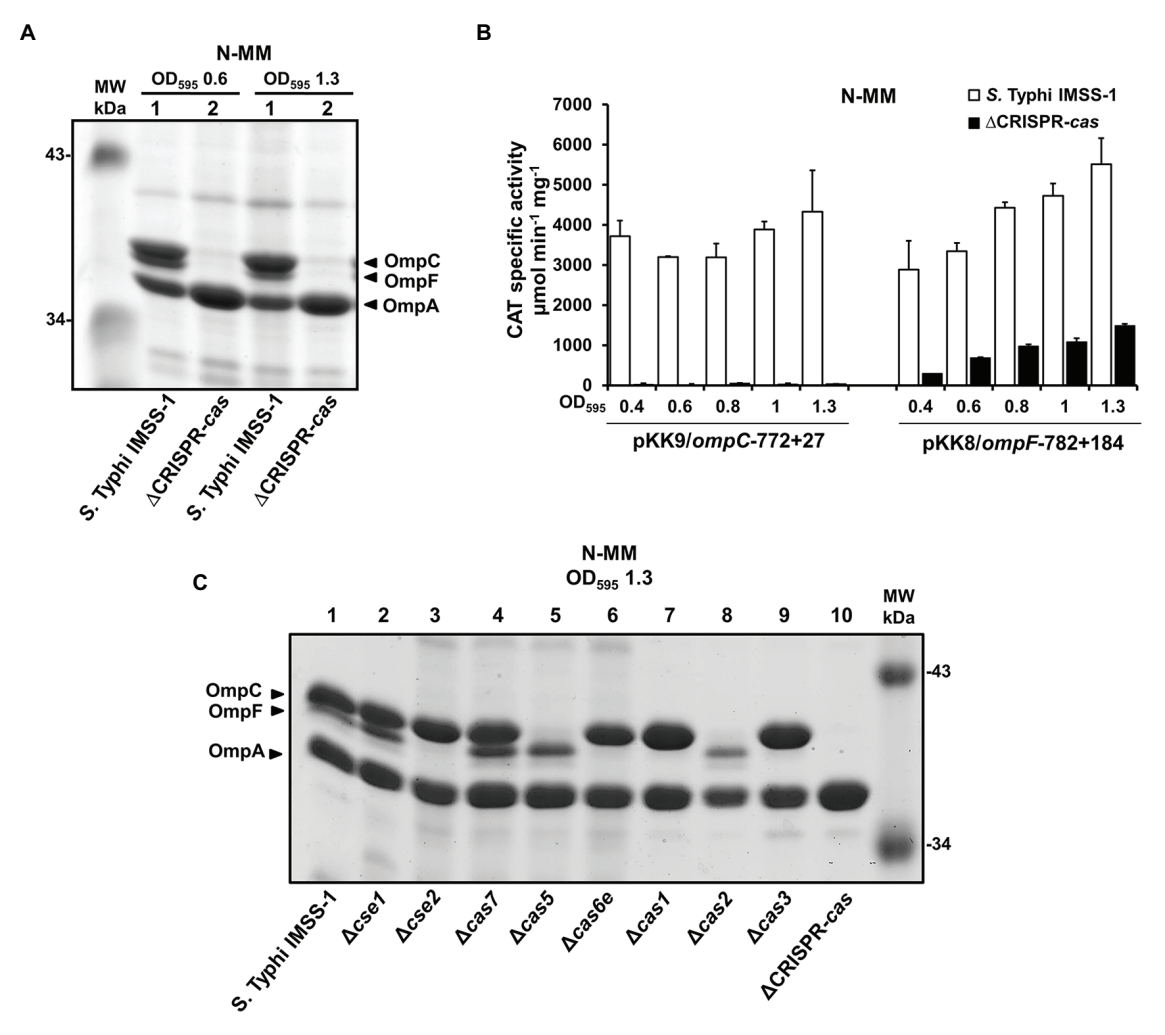
CRISPR-Cas is fundamental for the synthesis of the major outer membrane proteins OmpF and OmpC in *Salmonella* Typhi. **(A)** Electrophoretic pattern of Coomassie brilliant blue-stained outer membrane protein preparations, separated by 0.1% SDS-15% PAGE. The bacterial strains *Salmonella* Typhi IMSS-1 wild type (lane 1) and *Salmonella* Typhi ΔCRISPR-*cas* (ΔCRISPR-*cas*, lane 2) were grown in N-MM to an OD_595_ of 0.6 and 1.3. The major OMPs, OmpC, OmpF, and OmpA are indicated with a black triangle. **(B)** Transcriptional profiles of *Salmonella* Typhi IMSS-1 and *Salmonella* Typhi ΔCRISPR-*cas* harboring plasmid pKK9/*ompC*-772 + 27 or pKK8/*ompF*-782 + 184 in N-MM. CAT-specific activities were measured at an OD_595_ of 0.4, 0.6, 0.8, 1.0, and 1.3. The values are the means ± standard deviations for three independent experiments performed in duplicate. **(C)** Electrophoretic pattern of Coomassie brilliant blue-stained outer membrane protein preparations, separated by 0.1% SDS-15% PAGE from *Salmonella* Typhi IMSS-1 wild type (lane 1), Δ*cse1* (lane 2), Δ*cse2* (lane 3), Δ*cas7* (lane 4), Δ*cas5* (lane 5), Δ*cas6e* (lane 6), Δ*cas1* (lane 7), Δ*cas2* (lane 8), Δ*cas3* (lane 9), and *Salmonella* Typhi ΔCRISPR-*cas* (ΔCRISPR-*cas*, lane 10) strains, grown in N-MM at OD_595_ of 1.3. The OmpC, OmpF, and OmpA porins are indicated with a black triangle. Molecular weight markers (MW) are indicated.

Studies on the regulation and the signals that induce the CRISPR-*cas* locus in *S.* Typhi are available (Hernández-Lucas et al., 2008; Medina-Aparicio et al., 2011, 2017). However, its biological function in this human pathogen remains to be determined. In this regard, previous results in *Francisella novicida* demonstrated that the CRISPR-Cas system is involved in the synthesis of outer membrane proteins (Sampson et al., 2013, 2014). Therefore, we obtained a strain devoid of *cas3*, the *cas3-cse1* intergenic region, *cse1*, *cse2*, *cas7*, *cas5*, *cas6e*, *cas1*, *cas2*, and the CRISPR locus (the entire Type I-E CRISPR-Cas system, Figure 1), which was named as ΔCRISPR-*cas* (Supplementary Table S1). By electrophoretic profiles, the presence of the major outer membrane proteins OmpC, OmpF, and OmpA was detected in the wild-type strain; whereas in the isogenic *S.* Typhi strain devoid of CRISPR-*cas* locus, OmpC, and OmpF were not visualized (Figure 2A). To confirm these results, the transcriptional expression of *ompC* and *ompF* promoter regions was evaluated. Thus, the reporter plasmids pKK9/*ompC*-772 + 27 and pKK8/*ompF*-782 + 184 (Supplementary Table S1) were transformed into *S.* Typhi IMSS-1 wild type and, in the isogenic ΔCRISPR-*cas* strain, to perform CAT assays. The experiments showed that the transcriptional activity of the *ompC* and *ompF* regulatory regions were of 4,328 and 5,512 CAT units, respectively, in the wild-type strain. However, in the ∆CRISPR-*cas* strain the *ompC* and *ompF* activity decreased by 99 and 73%, respectively (Figure 2B). These data demonstrated that the CRISPR-Cas system is relevant for the expression of the major OmpC and OmpF porins in *S.* Typhi.

To determine the specific CRISPR-Cas genetic element involved in OmpC and OmpF regulation, a collection of individual *cas* mutants was generated, and porin profiles of these strains showed that Δ*cse1*, Δ*cas7*, and wild-type *S.* Typhi present a similar outer membrane protein profile. Nevertheless, in the absence of *cas5* and *cas2*, the amount of OmpC decreased substantially; whereas in the individual *cse2*, *cas6e*, *cas1* and *cas3* mutants the OmpF porin was not observed (Figure 2C). These data support the fundamental role of specific Cas proteins in the regulation of OmpC and OmpF major outer membrane proteins and also are in agreement with the results obtained from the deletion of the entire CRISPR-Cas locus, since this strain lacks *cas5*, *cas2*, *cse2*, *cas6e*, *cas1*, and *cas3*, which resulted in the absence of the two main porins in *S.* Typhi (Figure 2A).

**Figure 3:**
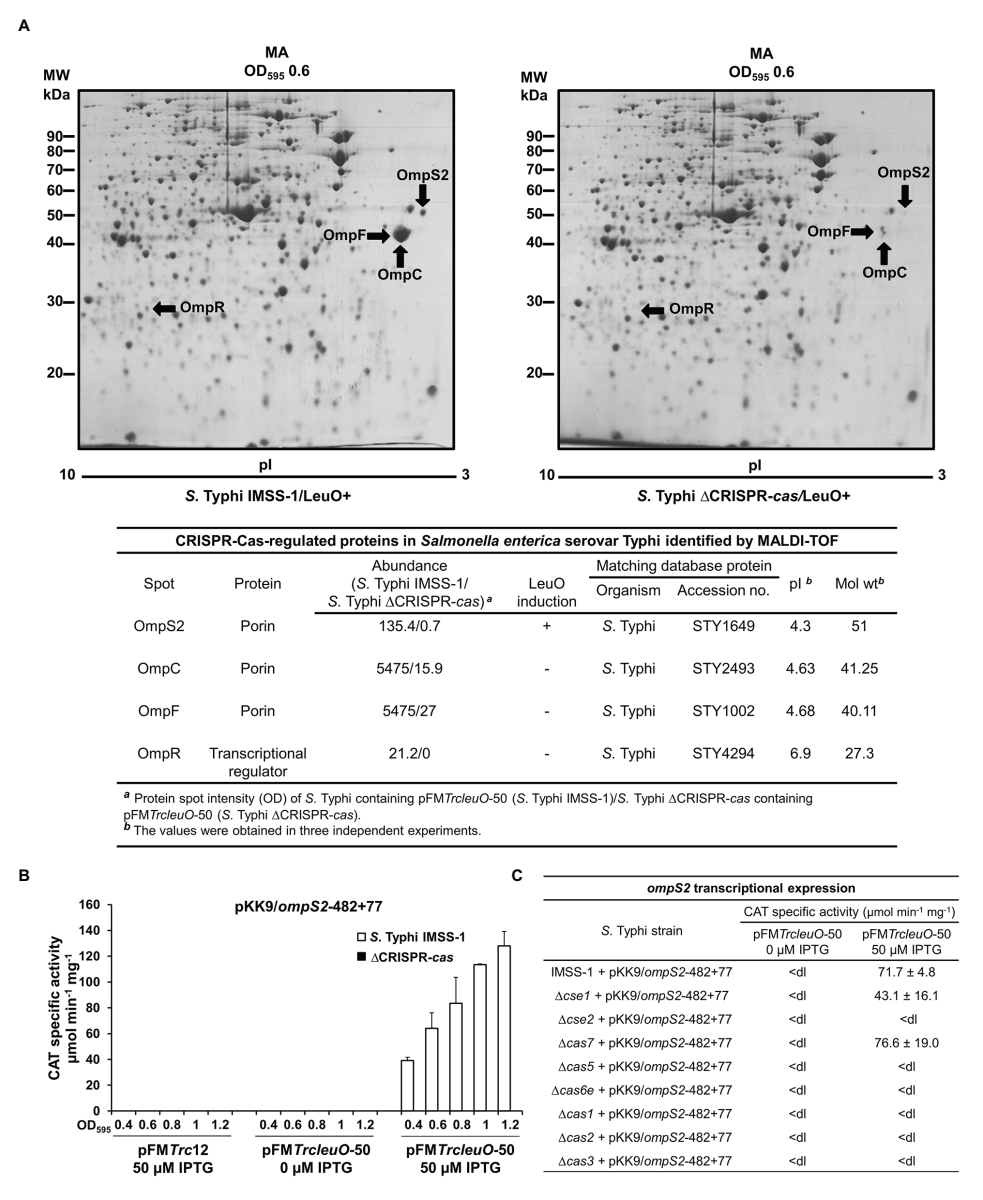
CRISPR-Cas is essential for the synthesis of Omps2, OmpC, and OmpF in *Salmonella* Typhi. **(A)** Proteomic profiles of *Salmonella* Typhi total protein extracts. *Salmonella* Typhi IMSS-1 wild type and *Salmonella* Typhi ΔCRISPR-*cas* containing pFM*TrcleuO*-50 were grown in MA to an OD_595_ of 0.6. Cultures were supplemented with 50 μM IPTG. The labeled spots were excised and identified using MALDI-TOF. Below the 2-DGE the CRISPR-Cas regulated proteins in *Salmonella* Typhi identified by MALDI-TOF are shown. **(B)** *ompS2* transcriptional activities. *Salmonella* Typhi IMSS-1 (white bars) and *Salmonella* Typhi ΔCRISPR-*cas* [ΔCRISPR-*cas*, black bars (values below the detection limit)] harboring plasmid pFM*TrcleuO*-50 or pFM*Trc*12 were independently transformed with pKK9/*ompS2*-482 + 77. The strains were grown in MA medium and CAT-specific activity was measured at OD_595_ of 0.4, 0.6, 0.8, 1.0, and 1.2. **(C)** Expression profiles of *Salmonella* Typhi IMSS-1, Δ*cse1*, Δ*cse2*, Δ*cas7*, Δ*cas5*, Δ*cas6e*, Δ*cas1*, Δ*cas2*, and Δ*cas3* strains containing pFM*TrcleuO*-50 and pKK9/*ompS2*-482 + 77 plasmids. The strains were grown in MA medium at OD_595_ of 1.0. The values are the means ± standard deviations for three independent experiments performed in duplicate; <dl (<detection limit) represents values between 0 and 10 CAT units.

To continue with the identification of more CRISPR-Cas dependent outer membrane proteins, and since the overexpression of LeuO induces quiescent porins, such as OmpS2 (Fernández-Mora et al., 2004), the induction of this protein was evaluated in the absence of CRISPR-Cas. *Salmonella* Typhi IMSS-1 harboring plasmid pFM*TrcleuO*-50 and *S.* Typhi ∆CRISPR-*cas* containing pFM*TrcleuO*-50 were grown to an OD_595_ of 0.6 in MA medium supplemented with IPTG (50 μM), and 2-DGE profiles were obtained with these cultures. The results showed the presence of OmpS2 in the wild-type strain. However, in the absence of the CRISPR-*cas* locus, OmpS2 decreased its expression by 99% (Figure 3A). Even more, the expression of a transcriptional fusion of the 5' intergenic region of *ompS2* (pKK9/*ompS2*-482 + 77, Supplementary Table S1), upon induction of the LeuO regulator at various points in the growth curve, was essentially abolished in the ∆CRISPR-*cas* as compared with the wild-type strain (Figure 3B). Therefore, CRISPR-Cas is also fundamental for OmpS2 expression mediated by LeuO.

To determine the specific *cas* genetic element involved in OmpS2 regulation, the individual *cas* mutants were transformed with the transcriptional CAT fusion containing the 5' regulatory region of *ompS2* and plasmid pFM*TrcleuO*-50 for overexpressing LeuO. The expression results showed that *ompS2* activity mediated by LeuO depends on *cse2*, *cas5*, *cas6e*, *cas1*, *cas2*, and *cas3*, since in the absence of each of these genetic elements *ompS2* was not transcribed (Figure 3C). These results indicated that the majority of Cas proteins, with exception of Cse1 and Cas7, are essential for the synthesis of the quiescent porin OmpS2. Thus, the presence of CRISPR-Cas cluster is essential for the synthesis of major and quiescent porins in *S.* Typhi.

### The *Salmonella* Typhi CRISPR-Cas System Is Involved in the Expression of the Porin Master Regulator OmpR

**Figure 4:**
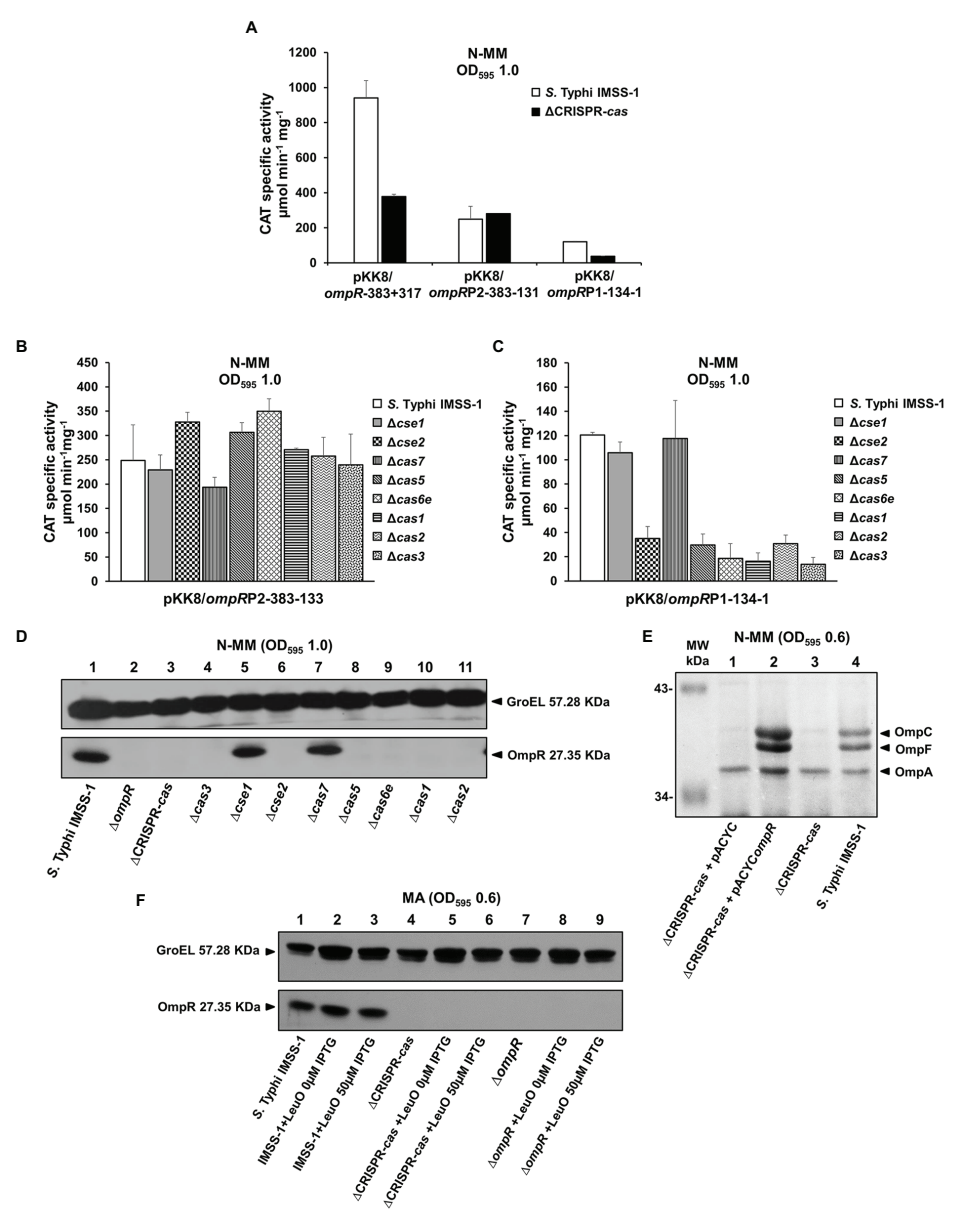
CRISPR-Cas is involved in the genetic control of OmpR. **(A)** Transcriptional profiles of *Salmonella* Typhi IMSS-1 and *Salmonella* Typhi IMSS-1 ΔCRISPR-*cas* (ΔCRISPR-*cas*) harboring plasmids pKK8/*ompR*-383 + 317, pKK8/*ompR*P2-383-133 (*ompR*P2) or pKK8/*ompR*P1-134-1 (*ompR*P1), grown in N-MM. CAT-specific activity was measured at an OD_595_ of 1.0. The values are the means ± standard deviations of three independent experiments performed in duplicate. The transcriptional expression of pKK8/*ompR*P2-383-133 **(B)** and pKK8/*ompR*P1-134-1 **(C)** was also evaluated in *Salmonella* Typhi IMSS-1, Δ*cse1*, Δ*cse2*, Δ*cas7*, Δ*cas5*, Δ*cas6e*, Δ*cas1*, Δ*cas2*, and Δ*cas3* strains grown in N-MM. The samples were collected at OD_595_ of 1.0. The values are the means ± standard deviations for three independent experiments performed in duplicate. **(D)** Western blot using anti-OmpR polyclonal antibody and total proteins from *Salmonella* Typhi IMSS-1 wild type (lane 1), *Salmonella* Typhi Δ*ompR* (Δ*ompR*, lane 2), *Salmonella* Typhi ΔCRISPR-*cas* (ΔCRISPR-*cas*, lane 3), Δ*cas3* (lane 4), Δ*cse1* (lane 5), Δ*cse2* (lane 6), Δ*cas7* (lane 7), Δ*cas5* (lane 8), Δ*cas6e* (lane 9), Δ*cas1* (lane 10), and Δ*cas2* (lane 11) strains were grown in N-MM at OD_595_ of 1.0. **(E)** Electrophoretic pattern of Coomassie brilliant blue-stained outer membrane protein preparations, separated by 0.1% SDS-15% PAGE of *Salmonella* Typhi ΔCRISPR-*cas* + pACYC (lane 1), *Salmonella* Typhi ΔCRISPR-*cas* + pACYC*ompR* (lane 2), *Salmonella* Typhi ΔCRISPR-*cas* (lane 3), and *Salmonella* Typhi IMSS-1 wild type (lane 4), grown in N-MM to an OD_595_ of 0.6. The major OMPs: OmpC, OmpF, and OmpA are indicated with a black triangle. **(F)** Western blot performed with anti-OmpR polyclonal antibody and total proteins from *Salmonella* Typhi IMSS-1 (lane 1), *Salmonella* Typhi IMSS-1 + pFM*TrcleuO*-50 (0 μM IPTG; lane 2), *Salmonella* Typhi IMSS-1 + pFM*TrcleuO*-50 (50 μM IPTG; lane 3), *Salmonella* Typhi IMSS-1 ∆CRISPR-*cas* (lane 4), *Salmonella* Typhi IMSS-1 ∆CRISPR-*cas* + pFM*TrcleuO*-50 (0 μM IPTG; lane 5), *Salmonella* Typhi IMSS-1 ∆CRISPR-*cas* + pFM*TrcleuO*-50 (50 μM IPTG; lane 6), *Salmonella* Typhi IMSS-1 ∆*ompR* (lane 7), *Salmonella* Typhi IMSS-1 ∆*ompR* + pFM*TrcleuO*-50 (0 μM IPTG; lane 8), *Salmonella* Typhi IMSS-1 ∆*ompR* + pFM*TrcleuO*-50 (50 μM IPTG, lane 9). All the *Salmonella* Typhi bacterial strains were grown in MA to an OD_595_ of 0.6. GroEL was used as protein loading control. The proteins visualized are indicated with black triangles.

The results mentioned above showed that CRISPR-Cas is involved in the synthesis of outer membrane proteins in *S.* Typhi. Interestingly, in the 2-DGE image shown in Figure 3A, a small spot of 27.3 kDa was absent in the ∆CRISPR-*cas*, and the mass spectrometry (MS) results of the same spot from *S.* Typhi IMSS-1, demonstrated that it corresponded to OmpR. To define whether CRISPR-Cas is involved in the control of the gene for this two-component system regulator, we evaluated its transcriptional expression in the *S.* Typhi wild type and in a ∆CRISPR-*cas*. The results showed that *ompR* displayed 941 CAT units in the wild-type strain, and the activity decreased by 60% in the CRISPR-Cas deficient *S.* Typhi strain (Figure 4A). Previously, it was demonstrated that *ompR* contains two promoters (Villarreal et al., 2014). To define whether the *ompR*P1 or *ompR*P2 promoters are under CRISPR-Cas control, the transcriptional activity of each promoter in the wild-type strain and in the ∆CRISPR-*cas* was evaluated. The transcriptional results showed *ompR*P2 activity values of 248 and 279 CAT units in the wild type and in the ∆CRISPR-*cas* isogenic strain, respectively (Figure 4A). Thus, CRISPR-Cas is not involved in *ompR*P2 promoter control. However, the activities obtained with *ompR*P1 were 120 and 37 CAT units in the wild type and in the ∆CRISPR-*cas*, respectively (Figure 4A). Therefore, CRISPR-Cas is involved in the regulation of the *ompR*P1 promoter to induce *ompR* expression.

To validate the results obtained and to determine the Cas proteins involved in *ompR*P1 genetic control, individual *cas* mutants were transformed with CAT fusions containing either the *ompR*P2 (pKK8/*ompR*P2-383-133) or the *ompR*P1 (pKK8/*ompR*P1-134-1) promoters. The activity results showed that in the *S.* Typhi wild type as well as in individual *cse1*, *cse2*, *cas7*, *cas5*, *cas6e*, *cas1*, *cas2*, and *cas3* null mutants, the *ompR*P2 promoter expression was similar, supporting the notion that the Cas proteins are not implicated in its regulation (Figure 4B). In the case of the *ompR*P1 promoter, its genetic activity in the individual *cse2*, *cas5*, *cas6e*, *cas1*, *cas2*, and *cas3* deficient strains was considerably reduced, compared with the CAT units obtained in the wild-type strain and in the *cse1* and *cas7* mutants (Figure 4C). The data support the proposal that the Cas proteins involved in *ompR*P1 promoter regulation correspond to Cse2, Cas5, Cas6e, Cas1, Cas2, and Cas3; whereas Cse1 and Cas7 are not implicated in *ompR*P1 induction.

To determine whether the reduction of *ompR*P1 promoter activity in the ∆CRISPR-*cas*, as well as in each *cas* individual mutant, has an effect on the synthesis of OmpR, western blot experiments were performed. The wild-type *S.* Typhi, the ∆CRISPR-*cas*, as well as the individual *cas3*, *cse1*, *cse2*, *cas7*, *cas5*, *cas6e*, *cas1*, and *cas2* deletion mutants were grown in N-MM to an OD_595_ of 1.0. Total crude cell protein extracts were transferred to membranes and probed using anti-OmpR polyclonal antibody. The western blot results showed a prominent OmpR band of 27.3 KDa in the wild-type strain and in the *cse1* and *cas7* individual mutants; whereas in the ∆CRISPR-*cas* strain, and in the individual *cse2*, *cas5*, *cas6e*, *cas1*, *cas2*, and *cas3* mutants the OmpR protein was absent (Figure 4D).

These results explain the lack of OmpC and OmpF in the corresponding *cas* deficient strains (Figure 2C), since it is well-known that OmpR binds to their regulatory regions to promote their expression (Yoshida et al., 2006). Therefore, *cse2*, *cas5*, *cas6e*, *cas1*, *cas2*, and *cas3* genes are fundamental for OmpR expression, whereas *cse1* and *cas7* are not involved in OmpR regulation, demonstrating that specific *cas* genes are necessary for OmpR production to control porin synthesis. Moreover, complementation of the *S.* Typhi ΔCRISPR-*cas* with the *ompR* gene on a plasmid restored the presence of OmpC and OmpF porins in this strain (Figure 4E), further supporting the notion that the deletion of the entire CRISPR-*cas* loci results in the lowering of the expression of the OmpR regulator and thus porin expression.

With respect to the OmpS2 quiescent porin, it is well accepted that LeuO counteracts the negative effect of H-NS on the *ompS2* promoter, upon which OmpR binds to its regulatory region promoting *ompS2* expression (Fernández-Mora et al., 2004). Thus, it was determined whether the OmpR protein was produced in the *S.* Typhi strains that overexpress LeuO. Western blot experiments demonstrated the presence of OmpR in *S.* Typhi IMSS-1 wild-type strain harboring the pFM*TrcleuO*-50 plasmid. However, OmpR was not detected in the *S*. Typhi ∆*cas*-CRISPR mutant overexpressing LeuO (Figure 4F). Thus, OmpS2 was not visualized in the 2-DGE of this strain (Figure 3A) because of the lack of the two-component system regulator OmpR.

In conclusion, the results obtained here showed that the CRISPR-Cas system acts hierarchically on the *ompR*P1 promoter to induce OmpC, OmpF, or OmpS2 synthesis in *S.* Typhi.

### The *Salmonella* Typhi *cas* Genes Are Involved in Sodium Deoxycholate Resistance and Biofilm Formation

**Figure 5:**
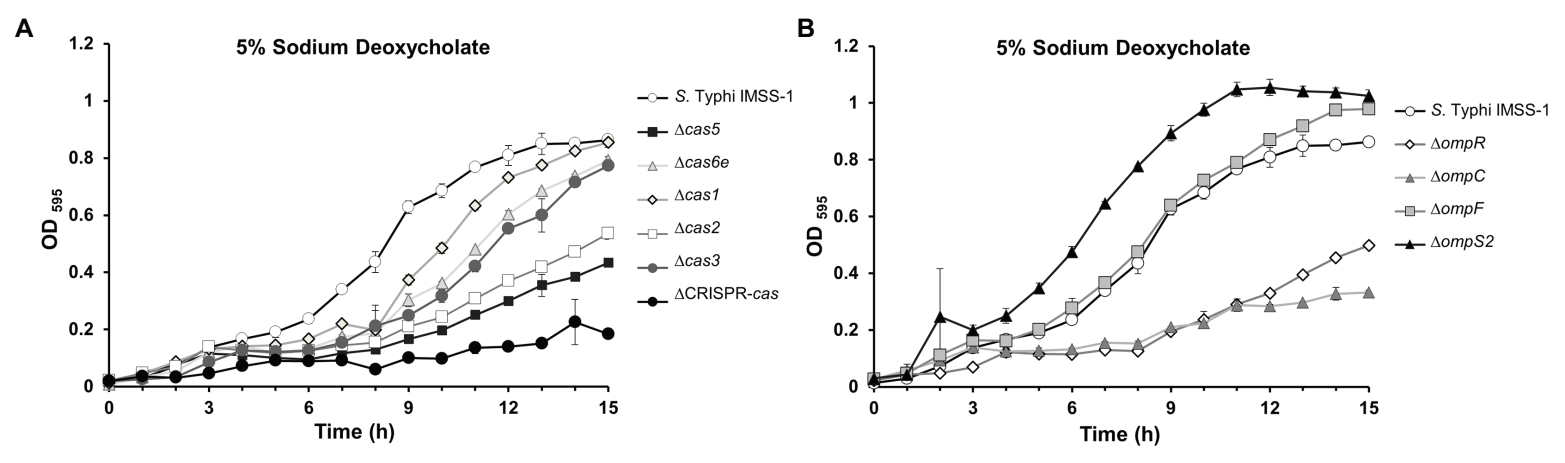
The CRISPR-Cas system is required for resistance to the bile salt sodium deoxycholate in *Salmonella* Typhi. **(A)** Growth kinetics in sodium deoxycholate of *Salmonella* Typhi IMSS-1 wild type (empty circle), Δ*cas5* (black square), Δ*cas6e* (gray triangle), Δ*cas1* (gray diamond), Δ*cas2* (empty square), Δ*cas3* (gray circle), and ΔCRISPR-*cas* (black circle). **(B)** Growth kinetics in sodium deoxycholate of *Salmonella* Typhi IMSS-1 wild type (empty circle), Δ*ompR* (empty diamond), Δ*ompC* (gray triangle), Δ*ompF* (gray square), and Δ*ompS2* (black triangle). For both kinetics, the bacterial strains were grown in LB broth supplemented with 5% sodium deoxycholate at 37°C. The growth was monitored by OD_595_. Three independent experiments were performed in duplicate and representative data are shown.

In this report, we have shown that the *S*. Typhi *cse2*, *cas5*, *cas6e*, *cas1*, *cas2*, and *cas3* genes are involved in porin synthesis through the regulation of the *ompR* gene which codes for the OmpR transcriptional regulator. In previous studies, it has been demonstrated that *ompR* is involved in virulence, sodium deoxycholate resistance, biofilm formation, the production of flagella, and curli (Pickard et al., 1994; Shin and Park, 1995; Vidal et al., 1998; Cameron and Dorman, 2012; Villarreal et al., 2014). Therefore, we evaluated whether the *cas* genes are involved in some of these biological processes. Growth rate experiments of *S*. Typhi IMSS-1 and the *cas* individual deleted strains were performed in LB broth supplemented with 5% of the human bile salt sodium deoxycholate. The results showed that the wild-type *S*. Typhi strain grew in this condition, reaching an OD_595_ of 0.86 after 15 h. However, growth of the *∆cas5*, ∆*cas2*, and *∆cas*-CRISPR mutant strains was impaired in the presence of this bile salt (Figure 5A) since their OD_595_ were of 0.43, 0.54, and 0.18, respectively, after 15 h of incubation. Remarkably, these strains did not express the OmpC porin, which was previously shown to be determinant for allowing *S.* Typhi to proliferate in the presence of sodium deoxycholate (Villarreal et al., 2014). The growth rate of ∆*cas6e*, ∆*cas1*, and ∆*cas3* was similar to that observed with the wild-type strain (Figure 5A), consistent with the presence of the OmpC porin in these mutants. The same experiment was performed with ∆*ompR*, ∆*ompC*, ∆*ompF*, and ∆*ompS2* strains. As expected, the *ompR* and *ompC* mutants were also impaired in their growth in 5% sodium deoxycholate (OD_595_ = 0.5 and 0.33, respectively; Figure 5B), as previously reported (Villarreal et al., 2014); whereas the ∆*ompF* and ∆*ompS2* mutant strains grew like the *S.* Typhi IMSS-1 wild type.

**Figure 6:**
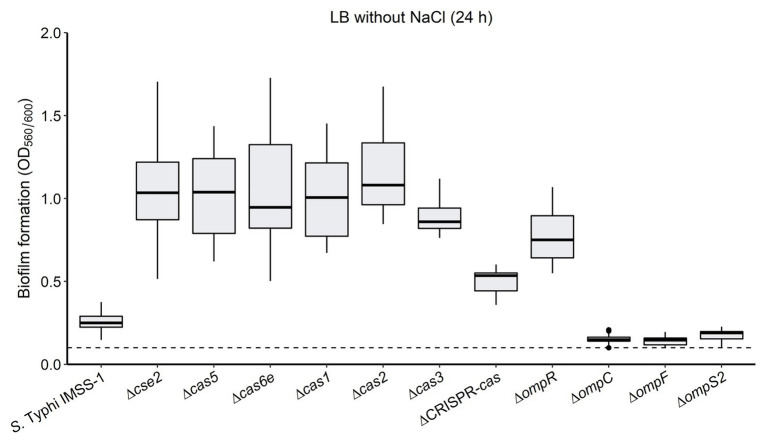
The absence of the CRISPR-Cas system increases biofilm formation in *Salmonella* Typhi. The *Salmonella* Typhi IMSS-1 wild type, and Δ*cse2*, Δ*cas5*, Δ*cas6e*, Δ*cas1*, Δ*cas2*, Δ*cas3*, ΔCRISPR-*cas*, Δ*ompR*, Δ*ompC*, Δ*ompF*, and Δ*ompS2* mutant strains were grown in LB broth without NaCl and incubated at 30°C for 24 h to evaluate biofilm formation by crystal violet staining in microtitre dishes. The dotted line indicates a 0.1 background value and a bacterial strain produces biofilm when the values obtained are above 0.1. The box plot diagram represents the data obtained from three independent experiments.

Additionally, we also evaluated the biofilm formation ability of the *S*. Typhi IMSS-1 wild type, and of the ∆*cse2*, ∆*cas5*, ∆*cas6e*, ∆*cas1*, ∆*cas2*, ∆*cas3*, *∆cas*-CRISPR, ∆*ompR*, ∆*ompC*, ∆*ompF*, and ∆*ompS2* mutant strains. The experiments showed that the wild type produced moderate biofilm (0.26 OD_560_/OD_600_ ratio). However, the *cse2*, *cas5*, *cas6e*, *cas1*, *cas2*, *cas3*, and *cas*-CRISPR mutants displayed an increased biofilm formation (OD_560_/OD_600_ ratio of 1.06, 1.04, 0.95, 1.04, 1.17, 0.90, and 0.50, respectively; Figure 6). These results suggest that the CRISPR-Cas system negatively regulates genes involved in biofilm production, i.e., that the absence of *cas* genes allows the expression of factors that increase the ability of *S.* Typhi to form biofilm.

The *∆ompR* mutant showed an increased biofilm formation (0.77), as compared to the values obtained with the wild type (0.26; Figure 6). However, strains carrying deletions either in the *ompC*, *ompF*, or the *ompS2* genes presented a slightly decreased biofilm formation (OD_560_/OD_600_ ratio of 0.15, 0.14, and 0.18, respectively; Figure 6). Therefore, the biofilm production was independent of the individual absence of the OmpC, OmpF, or OmpS2 porins. Interestingly, it has been demonstrated that *ompR* mutants in *Salmonella enteritidis*, *Salmonella pullorum*, *E. coli*, and *Yersinia enterocolitica* presented a decreased biofilm formation ability (Dong et al., 2011; Lu et al., 2012; Samanta et al., 2013; Meng et al., 2019), suggesting that the pathway toward regulating biofilm synthesis is different in *S*. Typhi.

The data shown are consistent with the notion that the CRISPR-Cas system is relevant for *S*. Typhi virulence, since this pathogen needs to survive the presence of bile salts in the gut and gallbladder, as well as to persist inside the gallbladder, where the biofilm formation is relevant (Crawford et al., 2010; Gonzalez-Escobedo et al., 2011; Spector and Kenyon, 2012).

## Discussion

The results presented here showed that the Cse2, Cas5, Cas6e, Cas1, Cas2, and Cas3 proteins *via* the positive regulation of the two-component regulator OmpR, have a role in the major and quiescent outer membrane protein synthesis, since they control OmpC, OmpF, and OmpS2. Due to the fact that only a few transcriptional factors have been implicated in the control of *ompR* in *Salmonella*, such as LtrR, H-NS, and OmpR (autoregulation; Bang et al., 2002; Villarreal et al., 2014), the data obtained contribute to the understanding of the regulatory network that controls the activity of this master regulator.

The results also support the complex genetic regulation of porins (De la Cruz and Calva, 2010), since in the absence of *cas5* and *cas2*, OmpR becomes undetectable (Figure 4D), as does OmpC (Figure 2C), demonstrating the specific role of these *cas* genes on *ompR* regulation to mediate OmpC synthesis. Interestingly, the presence of OmpF was evident in these *cas* mutants, supporting the notion that OmpF is not only OmpR-dependent, and that other transcriptional factors are able to induce OmpF expression. In this sense, regulators, such as Lrp and CadC, are also involved in its positive control (Ferrario et al., 1995; Lee et al., 2007; De la Cruz and Calva, 2010). In contrast, in the individual *cse2*, *cas6e*, *cas1*, and *cas3* mutants the OmpF porin was not visualized (Figure 2C), and OmpR was not detected by western blot (Figure 4D), supporting the role of these genes in the control of *ompR* to promote OmpF synthesis. In these *cas* mutants, the presence of OmpC was observed, supporting the proposal that other regulators are able to induce OmpC synthesis. In this respect, the CpxRA and CadC transcriptional factors have been reported to positively regulate *ompC* (Batchelor et al., 2005; Lee et al., 2007; De la Cruz and Calva, 2010).

In *E. coli*, it is well-known that *ompR-envZ* comprises an operon, and a bioinformatic analysis using the Operon-mapper tool suggested that, in *S.* Typhi, these genes can be also one transcriptional unit (data not shown; Taboada et al., 2018). Therefore, the absence of OmpR in the *cas* mutants indirectly suggests that EnvZ is not produced by the polar effect of the *ompR* deletion. However, OmpR is the principal component involved in porin synthesis since the presence of the corresponding porins was reestablished in the CRISPR-*cas* deleted strain overexpessing OmpR (Figure 4E).

In another report, it has also been shown that a Cas protein, Cas9, negatively regulates the gene coding for a transcriptional regulator of a two-component system: *regR*. In that case, it was demonstrated that Cas9 was able to degrade the *regR* mRNA, since the *Streptococcus agalactiae* CRISPR array contains two homologous sequences to the *regR* gene (Ma et al., 2018).

*Salmonella* Typhi contains a Type I-E CRISPR-Cas locus, and *in vitro* experiments have demonstrated that *E. coli* components of this genetic system are able to form a complex for recognition and degradation of viral and plasmid DNA (Brouns et al., 2008; Jore et al., 2011). The data obtained in this work showed that two Cas proteins, Cas5 and Cas2, are fundamental for OmpC expression, and other four Cas proteins, Cse2, Cas6e, Cas1, and Cas3, are required for OmpF synthesis. In the case of OmpS2 expression, six Cas proteins are relevant: Cse2, Cas5, Cas6e, Cas1, Cas2, and Cas3. Therefore, it is possible that different combinations of Cas form distinct protein complexes that bind, stabilize, and positively modulate the levels of *ompR* mRNA, for differentially regulating OmpC, OmpF, or OmpS2. Another possibility for OmpR regulation is that Cse2, Cas6e, Cas1, and Cas2 RNA-nucleases cleave the mRNA of a putative *ompR* repressor. Thus, when such negative regulator would be degraded, the *ompR* gene would be able to be expressed for porin synthesis. It is also possible that the function of Cas complexes would be only to bind at DNA to fine-tune *ompR* expression at specific promoters.

Currently, experiments are being performed in our laboratory to evaluate these hypotheses and to extend these initial observations in order to define how CRISPR-Cas mediate OmpR control. It is evident that much needs to be learned about the mechanisms by which various genetic elements control the expression of the OmpR regulator and thus, the porin phenotype in *S*. Typhi.

The finding that Cas proteins are able to regulate hierarchically the global two-component regulatory systems present in different proteobacteria, suggesting that the CRISPR-Cas systems could be involved in the regulation of biological processes controlled by two-component regulators, including oxidative stress, low pH, heat shock, bacterial motility, chemotaxis, osmotic changes, resistance to bile salts, and biofilm formation (Groisman, 2016; Pruss, 2017). In this sense, OmpR regulates the expression of *hilC*, *hilD*, and *ssrAB*, the main regulators of pathogenicity islands 1 and 2 of *Salmonella* Typhimurium, and it also controls the expression of the *viaB* locus that encodes Vi polysaccharide biosynthesis genes in *S*. Typhi (Pickard et al., 1994; Lee et al., 2000; Feng et al., 2003; Cameron and Dorman, 2012). Therefore, OmpR is implicated in regulation of virulence.

In the case of the OmpC and OmpF porins, a double mutant of these genes in *S.* Typhimurium was found to be attenuated for virulence in the mouse model (Chatfield et al., 1991). In addition, it has been observed that OmpC and OmpF induced long-term antibody response with bactericidal capacity and conferred protection against challenge with *S.* Typhi (Secundino et al., 2006; Pérez-Toledo et al., 2017). Moreover, it has been demonstrated that the immunization of mice with the OmpS2 protein induced the production of specific, long-term antibody titers and conferred protection against *S.* Typhi challenge. In addition, OmpS2 is a TLR2 and TLR4 agonist. Thus, OmpS2, despite being expressed at low levels under *in vitro* culture conditions, is a potent protective immunogen with intrinsic adjuvant properties (Moreno-Eutimio et al., 2013). *Salmonella* Typhimurium mutants with deletions in the *ompS2* gene were highly attenuated for virulence in a mouse model, supporting its role in pathogenesis (Rodríguez-Morales et al., 2006).

Thus, a phenotype for the mutants in the genes coding for the *S.* Typhi Cas was explored. It was found that the *cas5* and *cas2* genes are necessary for the optimal growth of *S*. Typhi in the presence of one of the major bile salts found in the human gut, sodium deoxycholate (Figure 5). Most remarkably, the ∆*cas5* and ∆*cas2* mutant strains lack the OmpC porin (Figure 2C), which was previously shown to be necessary for growth in the presence of this bile salt (Villarreal et al., 2014).

Additionally, the CRISPR-Cas system is implicated in the control of biofilm formation in *S*. Typhi, since the absence of *cse2*, *cas5*, *cas6e*, *cas1*, *cas2*, and *cas3* genes resulted in an increase in the biosynthesis of biofilm (Figure 6). Interestingly, the CRISPR-Cas system has also been involved in biofilm formation in *Pseudomonas aeruginosa* (Zegans et al., 2009). These newfound roles of the *S*. Typhi CRISPR-Cas system in the resistance to sodium deoxycholate and biofilm production should contribute toward the understanding of the evolutionary conservation of this system in the *Salmonella* genus, since these biological processes are relevant for the establishment of a successful infection cycle (Gonzalez-Escobedo et al., 2011; Spector and Kenyon, 2012).

Contributions from several other research groups also support the CRISPR-Cas-outer membrane protein association. By gene neighborhood analysis, it has been found that numerous candidate CRISPR-linked genes encode integral membrane proteins in bacterial and archaeal genomes (Shmakov et al., 2018). Furthermore, activation of the CRISPR-Cas system by envelope stress has been suggested in *E. coli* (Perez-Rodriguez et al., 2011), and a role in regulating the permeability of the bacterial envelope to resist membrane damage caused by antibiotics is suggested for CRISPR-Cas in *Francisella novicida* (Sampson et al., 2014). In *Myxococcus xanthus*, the CRISPR-Cas system appears to be involved in fruiting body development and exopolysaccharide production (Viswanathan et al., 2007; Wallace et al., 2014), Moreover, recent microarray experiments performed in our laboratory demonstrated that CRISPR-Cas is able to regulate other outer membrane encoded genes besides *ompC*, *ompF*, and *ompS2* (data not shown).

Collectively, these data, together with our results suggest a previously unappreciated role for CRISPR-Cas in the formation of bacterial structures and in the maintenance of the cell envelope in different prokaryotic organisms.

## Data Availability Statement

The raw data supporting the conclusions of this article will be made available by the authors, without undue reservation.

## Author Contributions

LM-A: methodology, formal analysis, investigation, writing-review, and editing. SR-G: methodology, formal analysis, and validation. JR-F: methodology and validation. AM-B, BM-M, EA-P, and AV: methodology. SE: methodology and resources. EC: writing-review and editing. IH-L: conceptualization, resources, writing-original draft preparation, visualization, supervision, project administration, and funding. All authors contributed to the article and approved the submitted version.

### Conflict of Interest

The authors declare that the research was conducted in the absence of any commercial or financial relationships that could be construed as a potential conflict of interest.

## Footnotes

^1^http://www.matrixscience.com
