# Supplementary material for: The CRISPR-Cas System Is Involved in OmpR Genetic Regulation for Outer Membrane Protein Synthesis in Salmonella Typhi
Source: Front Microbiol. 2021 Mar 29;12:657404. doi: 10.3389/fmicb.2021.657404 (PMC8039139; doi:10.3389/fmicb.2021.657404)
Supplement: Supplementary file 1 [file Table_1.docx]

**SUPPLEMENTARY MATERIAL**

**TABLE S1. Bacterial strains and plasmids**

| **Strain or plasmid** | **Genotype and/or relevant characteristics** | **Reference** |
| --- | --- | --- |
| ***S.* Typhi**  *S*. Typhi IMSS-1 | *Salmonella enterica* serovar Typhi 9.12, d, serotype; Mexican reference clinical strain. | Puente et al., 1987. |
| IMSS-1 + pFM*Trc12* | *S.* Typhi IMSS-1 containing the pFM*Trc12* plasmid, Ap^R^. | Hernández-Lucas et al., 2008. |
| IMSS-1 + pFM*TrcleuO*-50 | *S.* Typhi IMSS-1 containing the pFM*TrcleuO*-50 plasmid, Ap^R^. | Hernández-Lucas et al., 2008. |
| ∆*cas1* | *S.* Typhi IMSS-1 ∆*cas1* | This study |
| ∆*cas1* + pFM*Trc12* | *S.* Typhi IMSS-1 ∆*cas1* containing the pFM*Trc12* plasmid, Ap^R^. | This study |
| ∆*cas1* + pFM*TrcleuO*-50 | *S.* Typhi IMSS-1 ∆*cas1* containing the pFM*TrcleuO*-50 plasmid, Ap^R^. | This study |
| ∆*cas2* | *S.* Typhi IMSS-1 ∆*cas2* | This study |
| ∆*cas2* + pFM*Trc12* | *S.* Typhi IMSS-1 ∆*cas2* containing the pFM*Trc12* plasmid, Ap^R^. | This study |
| ∆*cas2* + pFM*TrcleuO*-50 | *S.* Typhi IMSS-1 ∆*cas2* containing the pFM*TrcleuO*-50 plasmid, Ap^R^. | This study |
| ∆*cas3* | *S.* Typhi IMSS-1 ∆*cas3* | This study |
| ∆*cas3* + pFM*Trc12* | *S.* Typhi IMSS-1 ∆*cas3* containing the pFM*Trc12* plasmid, Ap^R^. | This study |
| ∆*cas3* + pFM*TrcleuO*-50 | *S.* Typhi IMSS-1 ∆*cas3* containing the pFM*TrcleuO*-50 plasmid, Ap^R^. | This study |
| ∆*cas5* | *S.* Typhi IMSS-1 ∆*cas5* | This study |
| ∆*cas5* + pFM*Trc12* | *S.* Typhi IMSS-1 ∆*cas5* containing the pFM*Trc12* plasmid, Ap^R^. | This study |
| ∆*cas5* + pFM*TrcleuO*-50 | *S.* Typhi IMSS-1 ∆*cas5* containing the pFM*TrcleuO*-50 plasmid, Ap^R^. | This study |
| ∆*cas6e* | *S.* Typhi IMSS-1 ∆*cas6e* | This study |
| ∆*cas6e* + pFM*Trc12* | *S.* Typhi IMSS-1 ∆*cas6e* containing the pFM*Trc12* plasmid, Ap^R^. | This study |
| ∆*cas6e* + pFM*TrcleuO*-50 | *S.* Typhi IMSS-1 ∆*cas6e* containing the pFM*TrcleuO*-50 plasmid, Ap^R^. | This study |
| ∆*cas7* | *S.* Typhi IMSS-1 ∆*cas7* | This study |
| ∆*cas7* + pFM*Trc12* | *S.* Typhi IMSS-1 ∆*cas7* containing the pFM*Trc12* plasmid, Ap^R^. | This study |
| ∆*cas7* + pFM*TrcleuO*-50 | *S.* Typhi IMSS-1 ∆*cas7* containing the pFM*TrcleuO*-50 plasmid, Ap^R^. | This study |
| ∆CRISPR-*cas* | *S.* Typhi IMSS-1 ∆*cas3-cse1-cse2-cas7-cas5- cas6e-cas1-cas2*-CRISPR | Medina-Aparicio et al., 2011. |
| ∆CRISPR-*cas* + pFM*Trc*12 | *S.* Typhi IMSS-1 ∆CRISPR-*cas* harboring the pFM*Trc12* plasmid, Ap^R^. | This study |
| ∆CRISPR-*cas* + pFM*TrcleuO*-50 | *S.* Typhi IMSS-1 ∆CRISPR-*cas* containing the pFM*TrcleuO*-50 plasmid, Ap^R^. | This study |
| ∆*cse1* | *S.* Typhi IMSS-1 ∆*cse1* | This study |
| ∆*cse1* + pFM*Trc12* | *S.* Typhi IMSS-1 ∆*cse1* containing the pFM*Trc12* plasmid, Ap^R^. | This study |
| ∆*cse1* + pFM*TrcleuO*-50 | *S.* Typhi IMSS-1 ∆*cse1* containing the pFM*TrcleuO*-50 plasmid, Ap^R^. | This study |
| ∆*cse2* | *S.* Typhi IMSS-1 ∆*cse2* | This study |
| ∆*cse2* + pFM*Trc12* | *S.* Typhi IMSS-1 ∆*cse2* containing the pFM*Trc12* plasmid, Ap^R^. | This study |
| ∆*cse2* + pFM*TrcleuO*-50 | *S.* Typhi IMSS-1 ∆*cse2* containing the pFM*TrcleuO*-50 plasmid, Ap^R^. | This study |
| ∆*ompC* | IMSS-1 STYC171, *S.* Typhi IMSS-1 ∆*ompC,* Km^R^. | Martínez-Flores et al., 1999. |
| ∆*ompF* | IMSS-1 STYF302, *S.* Typhi IMSS-1 ∆*ompF,* Km^R^. | Martínez-Flores et al., 1999. |
| ∆*ompR* | *S*. Typhi IMSS-1 ∆*ompR.* | Villarreal et al., 2014. |
| ∆*ompR +*  pFM*Trc12* | *S.* Typhi IMSS-1 ∆*ompR* containing the pFM*Trc12* plasmid, Ap^R^. | This study |
| ∆*ompR* + pFM*TrcleuO*-50 | *S*. Typhi IMSS-1 ∆*ompR* containing the pFM*TrcleuO*-50 plasmid, Ap^R^. | This study |
| ∆*ompS2* | *S*. Typhi IMSS-1 ∆*ompS2.* Km^R^ | This study |
| ***E. coli***  DH5α | Φ80d/*lac*Z∆M15 ∆(*lac*ZYA-*arg*F) U169 *rec*A1*end*A1 *hsd*R17 (r_k_^-^m_k_^+^) *pho*A*sup*E44 λ-thi-*1 gyr*A96 *rel*A, Nal^R^. | Gibco BRL |
| **Plasmids** |  |  |
| pACYC | pACYC184 containing the p15A origin of replication, Cm^R^. | Chang and Cohen, 1978. |
| pFM*Trc*12 | p*Trc99*A derivative containing the p15A1 origin of replication, Ap^R^. | Hernández-Lucas et al., 2008. |
| pFM*TrcleuO*-50 | pFM*Trc12* derivative containing serovar Typhi *leuO* gene, behind an IPTG inducible P*trc* promoter, Ap^R^. | Hernández-Lucas et al., 2008. |
| pFMT*yompR* (pACYC*ompR*) | pACYC184 derivative containing the coding  region (720 pb) and 297 bp upstream of the  *S*. Typhi *ompR* gene, Cm ^R^. | Villarreal et al., 2014. |
| pKD4 | Plasmid containing the Km^R^ cassette for lambda Red recombination. | Datsenko and Wanner, 2000. |
| pKD46 | Red recombinase system under the arabinose promoter, Ap^R^. | Datsenko and Wanner, 2000. |
| pKK232-8 | pBR322 derivative containing a promoterless chloramphenicol acetyltransferase (*cat*) gene, Ap^R^. | Pharmacia LKB Biotechnology |
| pKK232-9 | pKK232-8 derivative, Km^R^. | Hernández-Lucas et al., 2008. |
| pKK8/*ompF*-782+184 | pKK232-8 derivative containing 782 bp upstream and 184 bp downstream of the *ompF* ATG start codon, Ap^R^. | Villarreal et al., 2014. |
| pKK8/*ompR*-383+317 | pKK232-8 derivative containing 383 bp upstream and 317 bp downstream of the *ompR* ATG start codon, Ap^R^. | This study |
| pKK8/*ompR*P1-134-1 | pKK232-8 derivative containing 134 bp upstream and -1 bp downstream of the *ompR* ATG start codon, Ap^R^. | Villarreal et al., 2014. |
| pKK8/*ompR*P2-383-133 | pKK232-8 derivative containing nucleotides from -383 to -133 regarding to the *ompR* ATG start codon, Ap^R^. | Villarreal et al., 2014. |
| pKK9/*ompC*-772+27 | pKK232-9 derivative containing 772 bp upstream and 27 bp downstream of the *ompC* ATG start codon, Km^R^. | Hernández-Lucas et al., 2008. |
| pKK9/*ompS2*-482+77 | pKK232-9 derivative containing 482 bp upstream and 77 bp downstream of the *ompS2* ATG start codon, Km^R^. | Hernández-Lucas et al., 2008. |

**TABLE S2. Oligonucleotides used in this study.**

| **Oligonucleotide** | **Sequence (5´-3´)** |
| --- | --- |
| *ompS1 Bam*HI-F | CGGGATCCCATAACGCTAATTTACCGCCCATGC |
| *ompS1 Kpn*I-R | GGGGTACCATTTGCTGCGCCTGCCACTAATAAC |
| *ompS2 Bam*HI-F | CGGGATCCAAGTGAGTCGACAGATTTTACCTGC |
| *ompS2 Kpn*I-R | GGGGTACCTTATAAATTTCAGCGGCGTGTGCTG |
| *ompC*/-772-748 *Bam*HI-F | CGGGATCCTAGAAGGGAGAATCGGGTAGAGACC |
| *ompC*/+27+3 *Kpn*I-R | GGGGTACCCAGGAGGGACAGTACTTTAACTTTC |
| *ompF*/-782-761 *Bam*HI-F | CGGGATCCGAAATGGGGCTGAATAAAGAGG |
| *ompF*/+184+163 *Kpn*I-R | GGGGTACCCAATCTGGGCATAAGTCTGGTC |
| *ompR*/-383-362 *Bam*HI-F | CGGGATCCTTTTTATTATACTGATAGTCGG |
| *ompR*/+317+299 *Bam*HI-F | CGGGATCCTTTTTATTATACTGATAGT |
| *ompR*/-133-162 *Kpn*I-R | GGGGTACCTACAATTAATCGGCAACCCCATTATTTATT |
| *ompR*/-134-110 *Bam*HI-F | CGGGATCCTATATTTAAGCTGCTGTTAAATATG |
| *ompR*/-1-24 *Kpn*I-R | GGGGTACCTGTCTGTACTCCCAAAGGTTCGCA |

**SUPPLEMENTARY REFERENCE**

Chang, A. C., and Cohen, S. N. (1978). Construction and characterization of amplifiable multicopy DNA cloning vehicles derived from the P15A cryptic miniplasmid. *J Bacteriol.* 134, 1141-1156. doi: 10.1128/JB.134.3.1141-1156.1978

Martínez-Flores, I., Cano, R., Bustamante, V. H., Calva, E., and Puente, J. L. (1999). The *ompB* operon partially determines differential expression of OmpC in *Salmonella* typhi and *Escherichia coli*. *J Bacteriol,* 181, 556–562. doi: 10.1128/JB.181.2.556-562.1999
